# Frontotemporal dementia and language networks: cortical thickness reduction is driven by dyslexia susceptibility genes

**DOI:** 10.1038/srep30848

**Published:** 2016-08-03

**Authors:** Donata Paternicó, Marta Manes, Enrico Premi, Maura Cosseddu, Stefano Gazzina, Antonella Alberici, Silvana Archetti, Elisa Bonomi, Maria Sofia Cotelli, Maria Cotelli, Marinella Turla, Anna Micheli, Roberto Gasparotti, Alessandro Padovani, Barbara Borroni

**Affiliations:** 1 Centre of Brain Aging, Neurology Unit, Department of Clinical and Experimental Sciences, University of Brescia, Brescia, Italy; 2 The III Laboratory, Biotechnology, Spedali Civili Hospital, Brescia, Italy; 3 Neurology Unit, Valle Camonica Hospital, Brescia, Italy; 4 IRCCS Centro San Giovanni di Dio Fatebenefratelli, Brescia, Italy; 5 Casa di Cura S. Francesco, Bergamo, Italy; 6 The Neuroradiology Unit, University of Brescia, Brescia, Italy

## Abstract

Variations within genes associated with dyslexia result in a language network vulnerability, and in patients with Frontotemporal Dementia (FTD), language disturbances represent a disease core feature. Here we explored whether variations within three related-dyslexia genes, namely *KIAA0319*, *DCDC2*, and *CNTNAP*, might affect cortical thickness measures in FTD patients. 112 FTD patients underwent clinical and neuropsychological examination, genetic analyses and brain Magnetic Resonance Imaging (MRI). *KIAA0319* rs17243157 G/A, *DCDC2* rs793842 A/G and *CNTNAP2* rs17236239 A/G genetic variations were assessed. Cortical thickness was analysed by Freesurfer. Patients carrying *KIAA0319* A*(AG or AA) carriers showed greater cortical thickness atrophy in the left fusiform and inferior temporal gyri, compared to *KIAA0319* GG (p ≤ 0.001). Patients carrying CNTNAP2 G*(*GA or GG*) showed reduced cortical thickness in the left insula then*CNTNAP2* AA carriers (p≤0.001). When patients with both *at-risk* polymorphisms were considered (*KIAA0319* A* and *CNTNAP*2 G*), greater and addictive cortical thickness atrophy of the left insula and the inferior temporal gyrus was demonstrated (p ≤ 0.001). No significant effect of *DCDC2* was found. In FTD, variations of *KIAA0319* and *CNTNAP2* genes were related to cortical thickness abnormalities in those brain areas involved in language abilities. These findings shed light on genetic predisposition in defining phenotypic variability in FTD.

Frontotemporal dementia (FTD) is a heterogeneous group of neurodegenerative disorders characterized by progressive deterioration of social behavior and personality, deficits of executive functions and language impairment. The different phenotypes within the FTLD spectrum include two major syndromes, namely the behavioral variant of frontotemporal dementia (bvFTD) mainly characterized by cognitive, personality and social comportment impairment; and the primary progressive aphasia (PPA), characterized by a fastest and predominant disorder of language^1^,^2^,^3^.

These phenotypes are characterized by frontal and temporal atrophy, thus language disorders manifestations are considered a core future of PPAs but present even in bvFTD^4^,^5^. They include abnormalities in speech production, word retrieval, object naming, word and sentence comprehension, as well as difficulty in the mastery of reading and/or spelling skills (dyslexia)^4^,^5^,^6^,^7^,^8^.

A high frequency of neurodevelopmental learning disability, including dyslexia, has been reported in FTD patients and their first-degree relatives^9^. Considering the strong genetic background of language acquisition as well as its impairment, it might be argue for the involvement of specific genes potentially causative of a susceptibility of language-associated brain regions in FTD^10^. Additionally, dyslexic individuals showed structural and functional changes of the left temporal regions, those regions selectively damaged in FTD patients^11^,^12^,^13^. Language-related genes might drive the anatomical distribution of regional atrophy in FTD and clinical presentation.

In this scenario, imaging-derived grey matter measurements could help to demonstrate whether those genes that predispose to language impairment modulate FTD presentation. In that regards, by voxel-based morphometry (VBM) methods, previous studies highlighted that *FOXP2* gene, linked to the acquisition of spoken language, is associated to greater hypoperfusion of the left frontal regions^14^,^15^. Similarly, we recently demonstrated that *KIAA0319* gene, associated to reading ability, is related to greater grey matter volume atrophy in the left temporal regions in FTD patients^16^. In the same study, no effect of other two dyslexia genes, namely *DCDC2* and *CNTNAP2*, was found by using VBM. However, previous findings underline the importance of selecting the appropriate neuroimaging measurement to successfully identify genes that influence brain structure or function^17^. More specifically, inimaging genetic studies it has been claimed that VBM may be limited by not discriminating genetically independent measurement as cortical thickness. Total grey matter volumes sum both cortical surface area and cortical thickness variables, these two measurements being demonstrated to depend on different genetic substrates^18^; thus, considering only one of these two variables, i.e. cortical thickness, might allow to obtain specific and reliable relationship with chosen genetic factors. Moreover, studies performed in a voxel-per-voxel basis generally require that the images are aligned to a common space, which has the potential for introducing additional biases, such as misalignment and misclassification^17^.

In the present study, we used a genetically independent and fully automated brain measure as cortical thickness to investigate the effect of three dyslexia genes, *KIAA0319*, *DCDC2*, *CNTNAP2* on language networks in a cohort of FTD patients, with the attempt to confirm and extend our previous VBM findings.

## Methods

### Setting and participants

Patients fulfilling criteria for FTD were consecutively recruited from the Centre for Neurodegenerative Disorders, University of Brescia, Italy, from December 2001 to July 2014. All patients underwent somatic and neurologic evaluation, routine laboratory examination, and a complete mental status evaluation. Only patients with brain MRI and blood sampling for genetic analyses were considered in the present study.

Each patient was screened for monogenic inherited disease, such as *GRN*, *MAPT*, or *C9orf72* mutations. Stringent exclusion criteria were applied as follows: (1) cerebrovascular disorders, previous stroke, hydrocephalus, and intracranial mass documented by MRI; (2) history of traumatic brain injury or another neurologic disease; (3) relevant medical problems (e.g., poorly controlled diabetes or hypertension; cancer within the past 5 years; clinically important hepatic, renal, cardiac, or pulmonary disorders); (4) history of major depressive disorder, bipolar disorder, schizophrenia, substance abuse disorder, or mental retardation according to DSM-IV criteria; (5) cerebrospinal fluid beta-amyloid/tau profile suggestive for Alzheimer disease (available in almost 60% of patients); and (6) logopenic variant of PPA (lvPPA), according to current clinical criteria^2^ as mainly associated with Alzheimer disease neuropathology^19^.

A comprehensive neuropsychological and behavioural assessment, including Basic Activities of Daily Living and Instrumental Activities of Daily Living, was carried out^20^,^21^. The neuropsychological testing was performed by a standardized neuropsychological battery including Mini-Mental State Examination, Frontotemporal Dementia-modified Clinical Dementia Rating scale (FTD-modified CDR), Raven Colored Progressive Matrices, Controlled Oral Word Association Test and Category Fluency, Clock Drawing Test, Rey Complex Figure Copy and Recall, Story Recall Test, Digit Span, Trail Making Test A and B, Token Test, and De Renzi Imitation Test^22^,^23^,^24^,^25^,^26^,^27^,^28^,^29^,^30^,^31^,^32^.

In addition, a language evaluation was further perform to patients with PPA. Behavioral disturbances were evaluated by using the Frontal Behavioral Inventory and Neuropsychiatry Inventory^33^,^34^.

Informed consent was obtained for blood collection and genetic analyses and for MRI scanning from each patient. The work was performed in accordance to the approved guidelines on human subjects research of Helsinki Declaration and it was approved by the local Ethics Committee of Brescia Hospital, Italy.

### Genetic analyses

Total genomic DNA was isolated from peripheral blood according to standard procedures. Three single nucleotide polymorphisms located within *KIAA0319/TTRAP/THEM2* locus (rs17243157 G/A), *DCDC2* (rs793842 A/G), and *CNTNAP2* (rs17236239 A/G) genes were evaluated. Primers for each polymorphism were reported in Supplementary Table 1.

The amplification protocols were designed as follows: 5 minutes at 95 °C for the first cycle, denaturation at 95 °C for 30 seconds, annealing ranging from 59 °C to 66 °C for 30 seconds (depending on the analyzed polymorphism), extension at 72 °C for 30 seconds for the subsequent 35 cycles, and a final extension at 72 °C for 5 minutes. The PCR products were analyzed on 2% agarose with 0.005% of ethidium bromide to reveal the reaction and to verify their size.

To assess rs17243157 and rs793842, denaturing high performance liquid chromatography (DHPLC) analysis on the WAVE nucleic acid fragment analysis system was performed (Transgenomic, Santa Clara, CA). Samples with an altered DHPLC profile were purified with Microcon Centrifugal filter devices (Amicon Bioseparations; Millipore Corp, Billerica, MA) and sequenced. Nucleotide direct sequencing was performed on genomic DNA for both strands by the ABI 3500xl DNA analyzer(Applied Biosystems, Foster City, CA) and analyzed using Chromas (Technelysium Pty Ltd, South Brisbane, Australia).

To assess rs17236239, direct sequencing was performed for both strands from purified PCR on the ABI 3500 DNA genetic analyzer (Applied Biosystems), according to the manufacturer’s instructions, and analyzed using Chromas (Technelysium Pty Ltd).

Genotype analyses were performed blinded to clinical diagnoses.

### MRI data acquisition and analyses

In the present study, brain images were collected using 2 different MRI scanners: (1) 1.5T MR scanner (Siemens Symphony, Erlangen, Germany), equipped with a circularly polarized transmit–receive coil to acquire 3D magnetization-prepared rapid gradient echo (MPRAGE) T1-weighted scan (repetition time [TR] 2.010 milliseconds, echo time [TE] 3.93 milliseconds, matrix 1 × 1 × 1, in-plane field of view [FOV] 250 3 250 mm^2^, slice thickness 1 mm, flip angle 15°); and (2) 1.5T MR scanner (Siemens Avanto) to acquire 3D MPRAGE T1-weighted scan (TR 2.050 milliseconds, TE 2.56 milliseconds, matrix 1 × 1 × 1, in-plane FOV 256 mm^2^, slice thickness 1 mm, flip angle 15°).

T1-weighted MRI were analyzed with FreeSurfer version 5.0 35. The surface-based pipeline consists of several stages previously described in detail^35^,^36^,^37^. Briefly, for each subject the T1-weighted, anatomical 3-dimensional MRI data sets were corrected for the intensity variations and a normalized intensity image was created. The volume was registered with the Talairach atlas through an affine registration. Next, the skull was stripped and extracerebral voxels were removed. Through a segmentation procedure, voxels were classified as white or grey matter, and cutting planes were chosen to separate the hemispheres. Cortical thickness measurements were than obtained by calculating the boundary between white matter and cortical grey matter in the left and right hemisphere separately at each of approximately 160,000 points per hemisphere across the cortical mantle.

To compare anatomic features across subjects, the surface of each subject was inflated to determine the large-scale folding patterns of the cortex and subsequently transformed into a sphere to minimize metric distortion. The folding patterns of each individual were then aligned with an average folding pattern using a high-resolution surface-based averaging. Thickness measures were mapped to the inflated surface of each participant’s brain reconstruction allowing visualization of data across the entire cortical surface. Finally, cortical thickness was smoothed with a 10-mm full width at half height Gaussian kernel to reduce local variations in the measurement.

### Statistical analysis

The data were analyzed by SPSS 21.0 software (http://www.spss.com). Subjects were analyzed according to genotype status for each evaluated polymorphism. Genotype distribution and allele frequencies were computed by Chi-Square test. Socio-demographic and clinical data were assessed by Mann-Whitney test and Chi-Square test, as appropriate.

Cortical thickness differences between groups were assessed using a vertex-by-vertex analysis and a two-sample *t* test in FreeSurfer, and the effect of each polymorphism was carried out by analysing patients carrying *at-risk* genotype vs. non carriers[i.e., KIAA0319 A*(*GA or AA*) vs KIAA0319 GG; DCDC2 G*(*GA or GG*) *vs*. DCDC2 AA; CNTNAP2 G*(*GA or GG*) *vs*. *CNTNAP2 AA]*. Moreover, the cumulative effect of *at-risk* polymorphisms was considered by analysing patients carrying all significant *at-risk* polymorphism vs. non-carriers.

Age, FTD-modified CDR, *GRN* autosomal dominant disorder (as the high number of patients bearing GRN mutation) and scanner type were introduced as covariates; the significance was set to *P* < 0.001 uncorrected for multiple comparisons, and cluster size was set to >300 vertices.

Finally, structural covariance analysis was further performed by means of FreeSurfer, to demonstrate that damaged areas were disconnected with intra- and inter- hemispheric brain regions. As previously demonstrated^38^, this approach relies on the assumption that functionally correlated brain regions show greater concordance in GM and WM volumes as a result of mutually trophic influences or common experience-related plasticity^39^,^40^. To this, a significant cluster of cortical thickness obtained in the previous analysis was selected and used as “seed” regions, exploring the pattern of covariance between the cortical thickness of this “seed” and the cortical thickness throughout the whole brain. More specifically the correlation analysis on a vertex-by-vertex basis was performed using the design matrix available in the Freesurfer - Qdec interface: a correlation model was estimated introducing as covariate of interest the thickness values of the selected seed region for each subjects. First the analysis was carried out in the three groups separately (no, one or two at-risk polymorphism carriers) to explore the pattern of covariance in FTD patients according to KIAA0319 and CNTNAP2 genotypes (p < 0.05 corrected for False Discovery Rate (FDR)). Then a statistical vertex-by-vertex direct comparisons of the connectivity pattern between groups was carried out (p ≤ 0.001 uncorrected).

## Results

### Subjects

One-hundred and eighteen subjects fulfilled inclusion/exclusion criteria. From this sample, 6 subjects were excluded for crashing of freesurfer pipeline and 112 entered in the present study, namely 79 patients with bvFTD, and 33 with PPA.

Demographic and clinical characteristics were reported in Table 1. No significant differences between bvFTD and PPA were found, with exception of gender.

No significant difference of genotype distribution and allele frequency of KIAA0319 (rs17243157 G/A), DCDC2 (rs793842 A/G) and CNTNAP2 (rs17236239 A/G) polymorphisms between bvFTD and PPA was reported (see Supplementary Table 2).

### MRI analyses

**Figure 1:**
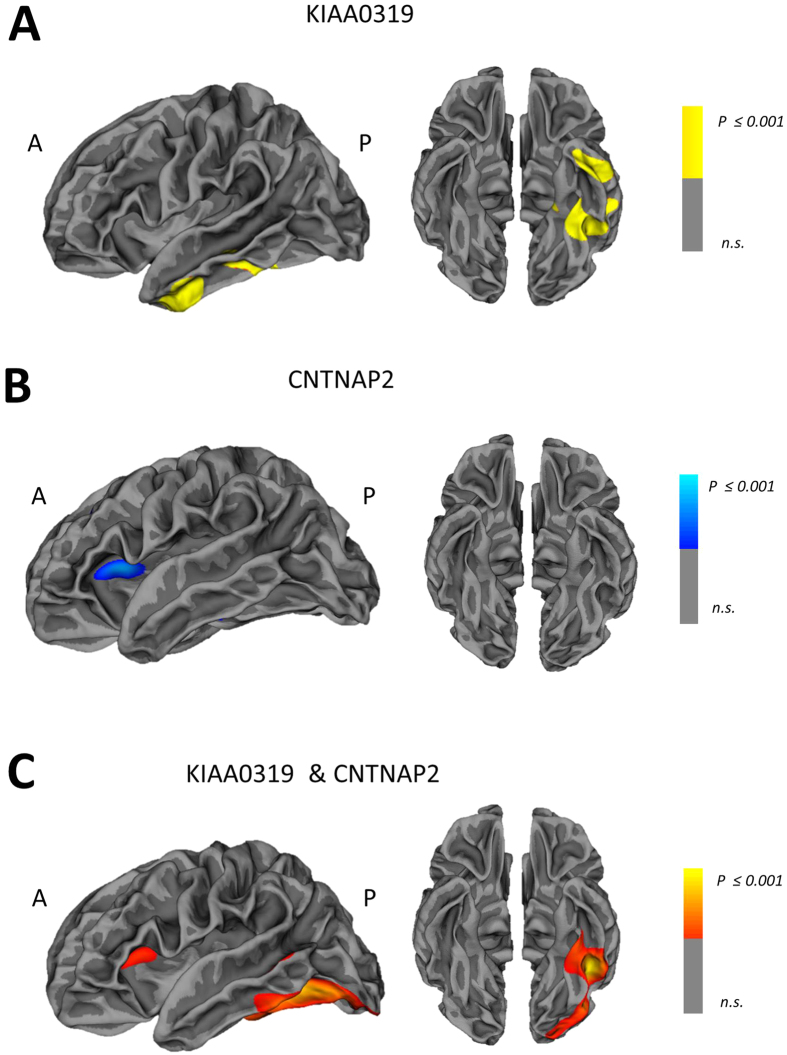
Cortical thickness reduction in FTD patients according to *KIAA0319and CNTNAP2* genotypes. Maps of cortical thickness reduction in FTD patients carrying only one at-risk genotype: *KIAA0319* A* vs. *KIAA0319* GG (A* < GG) (Panel A), *CNTNAP2 G* * *vs*. *CNTNAP2 AA* (G* < AA) (Panel B). Panel (C): maps of cortical thickness reduction in FTD patients carrying both at-risk genotypes (KIAA0319 A* and CNTNAP G*) vs. patients carrying only one *at-risk* polymorphism (KIAA0319 A* or CNTNAP G*) and non-carriers (KIAA0319 GG and CNTNAP AA). P ≤ 0.001 uncorrected, clusters ≥ 300 voxels. L = left; R = right.

As reported in Fig. 1A and Table 2, patients carrying *KIAA0319* A*(GA or AA) showed reduced cortical thickness in the left fusiform and inferior temporal gyri as compared to *KIAA0319* GG (p ≤ 0.0001).

As shown in Fig. 1B and Table 2, patients carrying *CNTNAP2* G*(GA or GG) showed reduced cortical thickness in the left insula as compared to *CNTNAP2* AA (p ≤ 0.001).

No significant differences in cortical thickness were found when DCDC2 (rs793842 A/G) was considered.

We then evaluated the cumulative effect of the two *at-risk* polymorphisms taken together (KIAA0319 A* and CNTNAP G*). Patients carrying both genetic variations showed greater cortical thickness atrophy of the left insula and inferior temporal gyrus again, in addition to the left middle temporal gyrus, as compared to patients with only one *at-risk* polymorphisms (KIAA0319 A* or CNTNAP G*) or none (KIAA0319 GG and CNTNAP AA) (p ≤ 0.001, see Fig. 1C and Table 2).

The inverse comparisons for each above analysis did not show any significant clusters above the pre-established threshold.

**Figure 2:**
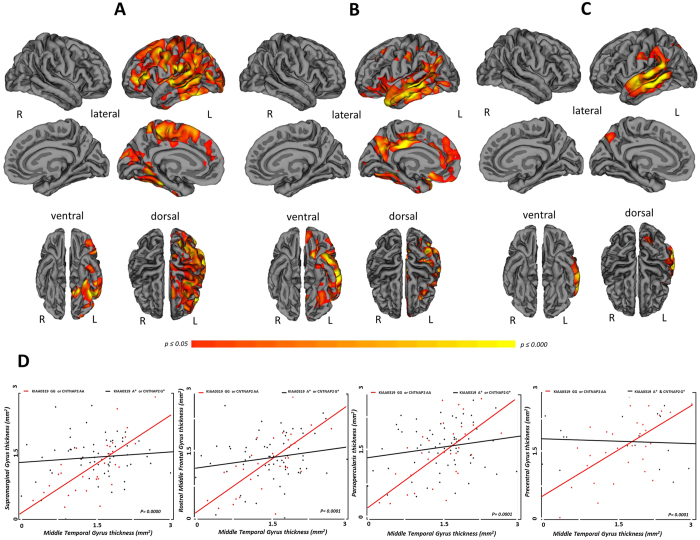
Cortical thickness structural connectivity analyses in FTD patients according to *KIAA0319 and CNTNAP2* genotypes. Pattern of structural correlation of the left middle temporal gyrus with other regions of the brain in: (Panel A) FTD patients carrying no *at-risk* polymorphism (KIAA0319 GG and CNTNAP AA), (Panel B) FTD patients carrying only one *at-risk* polymorphism (KIAA0319 A* or CNTNAP G*), (Panel C) FTD patients carrying both at-risk genotypes (KIAA0319 A* and CNTNAP G*). P ≤ 0.05 FDR corrected. L = left; R = right. Panel (D) Linear correlation of decreased structural association between brain regions in FTD patients carrying only one *at-risk* polymorphism (KIAA0319 A* or CNTNAP G*) or both at-risk genotypes (KIAA0319 A* and CNTNAP G*) (black lines) than patients carrying no *at-risk* polymorphism (red lines). P < 0.001 uncorrected.

To assess inter- and intra-hemispheric connectivity according to *KIAA0319 and CNTNAP2* genotypes, structural correlation analysis was carried out. As shown in Fig. 2, a progressive decreased structural correlation between the left middle temporal gyrus (peak MNI coordinates x, y, z: −55,−36,−10) and other intra-hemispheric brain regions was highlighted (p ≤ 0.05, FDR corrected). Patients with no *at-risk* polymorphism (KIAA0319 GG and CNTNAP AA) showed a widespread structural correlation of left temporal gyrus with the left hemisphere (Panel A); this correlation progressively worse in those patients carrying one *at-risk* polymorphism (KIAA0319 A* or CNTNAP G*) (Panel B), to patients carrying both *at-risk* polymorphisms (KIAA0319 A* and CNTNAP G*) (Panel C) (see also Supplementary Table 3a). Plots of the data in Panel D show the linear correlation of decreased structural association between brain regions in FTD patients carrying only one or both at-risk polymorphisms than patients carrying no at-risk genotype (see also Supplementary Table 3b).

## Discussion

FTD is a complex disorderthat encompassesheterogeneous phenotypes despite the same underlying neurodegenerative process^41^. The cause of this variability is not yet known. However, the well known genetic substrate of FTD might suggest that heritable factors interact with the neurodegenerative process and drive the anatomical distribution of damage.

Language impairment is a key feature of FTD^2^, and learning disabilities, including dyslexia, have been reported in FTD patients and their first degree relatives^19^. Consequently, it has been hypothesized that an antecedent genetic vulnerability leads to the involvement of brain language networks^10^. Recent genetic studies have corroborated this issue, and genetic variants linked to spoken language and reading abilities, i.e. *FOXP2*, *KIAA0319*, DYX1C1, DCDC2, have been demonstrated to modify grey/white matter volumes and activation, in healthy controls and in patients with dyslexia^42^,^43^,^44^,^45^,^46^.

In the same view, *FOXP2* and *KIAA0319* genetic variations have been associated with greater brain atrophy in the language areas in FTD patients^14^,^15^,^16^.

In the present study, we firstly assessed the effect of dyslexia-related genes by evaluating variations of cortical thickness on a wide cohort of FTD patients. Indeed, cortical thickness is considered a preferred measure, then gray matter volumes, to carefully identify how genetic background may influence brain structure^17^.

By using this approach, we showed that *KIAA0319* and *CNTNAP2* genetic variations affect cortical thickness in the left temporal regions. Moreover, bearing both genetic polymorphisms had an addictive effect on cortical thickness damage within language network areas. This was further suggested by the presence of an intra-hemispheric breakdown between damaged regions and the rest of the brain in FTD patients with both *at-risk* polymorphisms.

The genetic association with *KIAA0319* genotype confirmed and extended our previous voxel-based morphometry study^16^, and herein, by cortical thickness approach, we additionally provided evidence of *CNTNAP2* influence on language. Both *KIAA0319* and *CNTNAP2* are considered two of the most consistently replicated genes associated with dyslexia^47^. There are several evidence that variants in *KIAA0319* may contribute to reading abilities^48^,^49^,^50^,^51^, and previous genome-wide association studies have linked *CNTNAP2* to specific language impairment and dyslexia^52^,^53^,^54^. These genes are involved in neuronal migration and maturation during early development, and the genetic polymorphisms evaluated in the present study have been demonstrated to affect brain volume, brain connectivity and brain activation in language-related areas, and to drive hemispheric asymmetry^42^,^45^,^55^,^56^.

Indeed, temporal regions and perisylvian areas, including insula, represent an important neural correlate of dyslexia^57^,^58^,^59^,^60^.

In conclusion, we demonstrated that selective cortical thickness reduction in language-related areas is driven by dyslexia susceptibility genes in FTD patients. These results argue for the relevant role of language-related genes in modulating the clinical phenotype.

We acknowledge that this study entails some limits. Statistical analysis were not corrected for multiple comparisons and neuropathological confirmation was missing, and further studies considering only Primary Progressive Aphasia subtypes and Alzheimer dementia, are warranted. Furthermore, we have no reliable information on learning disabilities in these patients and in their siblings.

New development in molecular genetics have a great potential for characterizing speech and language impairments and for understanding their etiology and developmental course in the neurodegenerative conditions.

## Additional Information

**How to cite this article**: Paternicó, D. *et al*. Frontotemporal dementia and language networks: cortical thickness reduction is driven by dyslexia susceptibility genes. *Sci. Rep*. **6**, 30848; doi: 10.1038/srep30848 (2016).

## Supplementary Material

Supplementary Table S1

Supplementary Table S2

Supplementary Table S3

## Figures and Tables

**Table 1:** Demographic and clinical characteristics of FTD patients.

	*All FTD* (*n* = *112*)	bvFTD (n = 79)	PPA (n = 33)	*p*-value^
Age atevaluation, years	65.2 ± 7.6	65.1 ± 6.9	66.1 ± 9.2	0.99
Education, years	8 ± 3.6	8.0 ± 3.6	8.0 ± 3.4	0.96
Gender, M (%)	58 (51.8)	47 (59.5)	11 (33.3)	0.01*
Handedness, right(%)	109 (97)	76 (96)	33 (100)	0.42*
GRN mutation, n (%)	22 (20)	14 (18)	8 (24)	0.29
FTD-modified CDR	5.1 ± 3.4	5.2 ± 3.6	4.8 ± 3.0	0.54
FBI	15.7 ± 9.8	17.4 ± 10.5	11.5 ± 6.2	0.003
BADL	0.42 ± 1.0	0.49 ± 1.1	0.27 ± 0.9	0.32
IADL	1.46 ± 2.1	1.45 ± 2.0	1.48 ± 2.3	0.95

FTD: Frontotemporal Dementia; bvFTD: behavioral variant of Frontotemporal Dementia; PPA: Primary Progressive Aphasia; M: males; GRN: Granulin; FTD-modified CDR: Frontotemporal Dementia-modified Clinical Dementia Rating scale; FBI: Frontal Behavioral inventory; IADL: Instrumental Activities of Daily Living; BADL: Basic Activities of Daily Living. Results are expressed as mean ± standard deviation; percentage between brackets. ^Student-T test, otherwise specified; *Chi-Square test.

**Table 2:** Cortical thickness differences according to KIAA0319 and CNTNAP2 genotypes.

	Region	Side	K	Coordinates (x, y, z)	Mean Values		*p*
*KIAA0319*					**GG**	A*	
	Fusiform Gyrus	L	535	−41 −44 −17	2.25 ± 0.32	2.13 ± 0.44	0.000
	Inferior Temporal Gyrus	L	566	−47 −8 −33	2.54 ± 0.46	2.47 ± 0.48	0.000
*CNTNAP2*					**AA**	G*	
	Insula	L	444	−30 14 7	2.25 ± 0.25	2.19 ± 0.24	0.001
*KIAA0319 & CNTNAP2*					**0 or 1** ***at-risk*** **SNP**	**2** ***at-risk*** **SNPs**	
	Inferior Temporal Gyrus	L	4335	−47 −50 −14	2.34 ± 0.48	2.27 ± 0.30	0.000
	Middle Temporal Gyrus	L	1162	−55 −36 −10	2.09 ± 0.33	2.01 ± 0.52	0.001
	Insula	L	749	−29 19 9	2.56 ± 0.32	2.36 ± 0.43	0.001

Coordinates denote the peak voxels of each cluster in standard space, K = cluster size (number of contiguous significant vertices), Side: L = left; R = right. Mean values denote the average cortical thickness values and SD in the cluster for each group.
