# Supplementary material for: Frontotemporal dementia and language networks: cortical thickness reduction is driven by dyslexia susceptibility genes
Source: Sci Rep. 2016 Aug 3;6:30848. doi: 10.1038/srep30848 (PMC4971514; doi:10.1038/srep30848)
Supplement: Supplementary Table S1 [file srep30848-s1.docx]

**Frontotemporal dementia and language networks: cortical thickness reduction is driven by dyslexia susceptibility genes**

Donata Paternicó^1^, MS; Marta Manes, MD^2^; Enrico Premi, MD^2^; Maura Cosseddu, MS^2^; Stefano Gazzina, MD^2^; Antonella Alberici, MD^2^, Silvana Archetti, PhD^3^; Elisa Bonomi, MS^2^; Maria Sofia Cotelli, MD^4^; Maria Cotelli, MS^6^; Marinella Turla, MD^4^; Anna Micheli, MD^5^; Roberto Gasparotti, MD^7^; Alessandro Padovani, MD, PhD^2^; Barbara Borroni, MD^2^*

1 Centre of Brain Aging, Neurology Unit, Department of Biomedical Sciences and Translational Medicine, University of Brescia, Brescia, Italy;

2 Centre of Brain Aging, Neurology Unit, Department of Clinical and Experimental Sciences, University of Brescia, Brescia, Italy;

3 the III Laboratory, Biotechnology, Spedali Civili Hospital, Brescia, Italy;

4 Neurology Unit, Valle Camonica Hospital, Brescia, Italy;

5 Casa di Cura S. Francesco, Bergamo, Italy;

6 IRCCS Centro San Giovanni di Dio Fatebenefratelli, Brescia, Italy;

7 the Neuroradiology Unit, University of Brescia, Brescia, Italy.

**Table 1. Forward and reverse primers designed for testing single nucleotide polymorphisms (SNPs) within *KIAA0319/TTRAP/THEM2 locus, DCDC2 and CNTNAP2* genes.**

| **SNP** | **Primer** |
| --- | --- |
|  |  |
| rs17243157 F | 5’-ACGAACTTCGGAAGAGCTGGAA-3’ |
| rs17243157 R | 5’-GAAATGCGCTGCTCTCGGAA-3’ |
| rs793842 F | 5’-GGCAGATGTCTATTAGACCATGA-3’ |
| rs793842 R | 5’-TGACAGTCAGAGGTGGTG-3’ |
| rs17236239 F | 5’-CTTGATATGAGACCTCAGGTTTTGAGC-3’ |
| rs17236239 R | 5’-AACCCAATTTGATGAAGACACGACC -3' |

SNP: single nucleotide polymorphism; F: forward; R: reverse.
