# Supplementary material for: Frontotemporal dementia and language networks: cortical thickness reduction is driven by dyslexia susceptibility genes
Source: Sci Rep. 2016 Aug 3;6:30848. doi: 10.1038/srep30848 (PMC4971514; doi:10.1038/srep30848)
Supplement: Supplementary Table S2 [file srep30848-s2.docx]

**Frontotemporal dementia and language networks: cortical thickness reduction is driven by dyslexia susceptibility genes**

Donata Paternicó^1^, MS; Marta Manes, MD^2^; Enrico Premi, MD^2^; Maura Cosseddu, MS^2^; Stefano Gazzina, MD^2^; Antonella Alberici, MD^2^, Silvana Archetti, PhD^3^; Elisa Bonomi, MS^2^; Maria Sofia Cotelli, MD^4^; Maria Cotelli, MS^6^; Marinella Turla, MD^4^; Anna Micheli, MD^5^; Roberto Gasparotti, MD^7^; Alessandro Padovani, MD, PhD^2^; Barbara Borroni, MD^2^*

1 Centre of Brain Aging, Neurology Unit, Department of Biomedical Sciences and Translational Medicine, University of Brescia, Brescia, Italy;

2 Centre of Brain Aging, Neurology Unit, Department of Clinical and Experimental Sciences, University of Brescia, Brescia, Italy;

3 the III Laboratory, Biotechnology, Spedali Civili Hospital, Brescia, Italy;

4 Neurology Unit, Valle Camonica Hospital, Brescia, Italy;

5 Casa di Cura S. Francesco, Bergamo, Italy;

6 IRCCS Centro San Giovanni di Dio Fatebenefratelli, Brescia, Italy;

7 the Neuroradiology Unit, University of Brescia, Brescia, Italy.

**Table 2. Genotype distribution and allele frequencies in FTD patients.**

|  | ***Genotype distribution*** | | | |  |  | ***Allele frequency*** | | |
| --- | --- | --- | --- | --- | --- | --- | --- | --- | --- |
| ***Genotype*** |  | ***bvFTD*** | ***PPA*** | ***P- value*** |  | ***Allele*** | ***bvFTD*** | ***PPA*** | ***P-value*** |
| ***KIAA0319*** | **GG** | 78.5 (62) | 75.8 (25) |  |  | **G** | 88.0 (139) | 86.4 (57) | 0.74 |
|  | **AG** | 19 (15) | 21.2 (7) | 0.95 |  | **A** | 12.0 (19) | 13.6 (9) |  |
|  | **AA** | 2.5 (2) | 3.0 (1) |  |  |  |  |  |  |
| ***DCDC2*** | **GG** | 41.8 (33) | 39.4 (13) |  |  | **G** | 61.4 (97) | 63.6 (42) | 0.75 |
|  | **AG** | 39.2 (31) | 48.5 (16) | 0.56 |  | **A** | 38.6 (61) | 36.4 (24) |  |
|  | **AA** | 19 (15) | 12.1 (4) |  |  |  |  |  |  |
| ***CNTNAP2*** | **GG** | 17.7 (14) | 15.2 (5) |  |  | **G** | 42.4 (67) | 37.9 (25) | 0.53 |
|  | **AG** | 49.4 (39) | 45.5 (15) | 0.80 |  | **A** | 57.6 (91) | 62.1 (41) |  |
|  | **AA** | 32.9 (26) | 39.4 (13) |  |  |  |  |  |  |

bvFTD: behavioral variant of Frontotemporal Dementia; PPA: Primary Progressive Aphasia. Results are expressed as percentage and numbers between brackets.
