# Supplementary material for: Frontotemporal dementia and language networks: cortical thickness reduction is driven by dyslexia susceptibility genes
Source: Sci Rep. 2016 Aug 3;6:30848. doi: 10.1038/srep30848 (PMC4971514; doi:10.1038/srep30848)
Supplement: Supplementary Table S3 [file srep30848-s3.docx]

**Frontotemporal dementia and language networks: cortical thickness reduction is driven by dyslexia susceptibility genes**

Donata Paternicó^1^, MS; Marta Manes, MD^2^; Enrico Premi, MD^2^; Maura Cosseddu, MS^2^; Stefano Gazzina, MD^2^; Antonella Alberici, MD^2^, Silvana Archetti, PhD^3^; Elisa Bonomi, MS^2^; Maria Sofia Cotelli, MD^4^; Maria Cotelli, MS^6^; Marinella Turla, MD^4^; Anna Micheli, MD^5^; Roberto Gasparotti, MD^7^; Alessandro Padovani, MD, PhD^2^; Barbara Borroni, MD^2^*

1 Centre of Brain Aging, Neurology Unit, Department of Biomedical Sciences and Translational Medicine, University of Brescia, Brescia, Italy;

2 Centre of Brain Aging, Neurology Unit, Department of Clinical and Experimental Sciences, University of Brescia, Brescia, Italy;

3 the III Laboratory, Biotechnology, Spedali Civili Hospital, Brescia, Italy;

4 Neurology Unit, Valle Camonica Hospital, Brescia, Italy;

5 Casa di Cura S. Francesco, Bergamo, Italy;

6 IRCCS Centro San Giovanni di Dio Fatebenefratelli, Brescia, Italy;

7 the Neuroradiology Unit, University of Brescia, Brescia, Italy.

**Table 3a.** **Cortical thickness structural correlation in patients groups according to KIAA0319 and CNTNAP2 .**

|  | **Region** | **Side** | **K** | **Coordinates (x, y, z)** | ***P (FDR corrected)*** |
| --- | --- | --- | --- | --- | --- |
| ***KIAA0319 GG or CNTNAP2 AA*** | Middle Temporal Gyrus | L | 70060 | -54 -36 -10 | 0.000 |
|  | Isthmus Cingulate | L | 734 | -11 -52 7 | 0.000 |
|  | Lingual Gyrus | L | 661 | -8 -51 -18 | 0.000 |
|  | Precuneus | L | 1063 | 61 -51 -7 | 0.000 |
|  | Superior Parietal Gyrus | L | 636 | 58 -46 -23 | 0.002 |
|  | Inferior Temporal | L | 602 | -42 -6 -41 | 0.003 |
|  | Superior Frontal Gyrus | L | 481 | 26 43 -9 | 0.008 |
|  | Rostral Anterior Cingulate Gyrus | L | 63 | 8 35 -6 | 0.02 |
|  |  |  |  |  |  |
|  | Middle Temporal Gyrus | L | 31982 | -54 -36 -10 | 0.000 |
| ***KIAA0319 A* or CNTNAP2 G**** | Lateral Orbitofrontal Gyrus | L | 2810 | -16 -16 -15 | 0.000 |
|  | Posterior Cingulate Gyrus | L | 12896 | -5 -27 34 | 0.000 |
|  | Postcentral Gyrus | L | 1224 | -55 -20 38 | 0.000 |
|  | Rostral Middle Frontal | L | 753 | -28 30 36 | 0.002 |
|  | Superior Parietal Gyrus | L | 281 | -31 -42 52 | 0.003 |
|  | Parahippocampal Gyrus | L | 46 | -15 -39 -8 | 0.006 |
|  |  |  |  |  |  |
|  | Middle Temporal Gyrus | L | 13600 | -55 -37 -10 | 0.000 |
|  | Superior Parietal Gyrus | L | 2378 | -19 -63 41 | 0.000 |
|  | Postcentral Gyrus | L | 683 | -59 -15 30 | 0.000 |
| ***KIAA0319 A* & CNTNAP2 G**** | Precentral Gyrus | L | 369 | -49 3 15 | 0.002 |
|  | Inferior Parietal Gyrus | L | 137 | 65 -42 -54 | 0.004 |
|  | Precuneus | L | 55 | 20 -15 -44 | 0.004 |
|  | Parahippocampal Gyrus | L | 19 | -25 -43 -9 | 0.004 |

**Table 3b. Cortical thickness structural correlation differences between groups.**

|  | **Region** | **Side** | **K** | **Coordinates (x, y, z)** | ***P (uncorrected)*** |
| --- | --- | --- | --- | --- | --- |
|  |  |  |  |  |  |
| ***KIAA0319 GG or CNTNAP2 AA***  ***Vs*** | Supramarginal Gyrus | L | 637 | -56 -21 23 | 0.000 |
| ***vs*** | Rostral Middle Frontal Gyrus | L | 208 | -39 45 4 | 0.001 |
| ***KIAA0319 A* or CNTNAP2 G**** | Parsopercularis | L | 539 | -44 22 19 | 0.001 |
|  |  |  |  |  |  |
|  |  |  |  |  |  |
| ***KIAA0319 GG or CNTNAP2 AA***  ***Vs*** |  |  |  |  |  |
| ***Vs*** | Precentral Gyrus | L | 118 | -57 0 35 | 0.001 |
| ***KIAA0319 A* & CNTNAP2 G**** |  |  |  |  |  |

Coordinates denote the peak voxels of each cluster in standard space, K= cluster size (number of contiguous significant vertices), L= left; R =right.
